# Modified Mahalanobis Taguchi System for Imbalance Data Classification

**DOI:** 10.1155/2017/5874896

**Published:** 2017-07-24

**Authors:** Mahmoud El-Banna

**Affiliations:** Industrial Engineering Department, German Jordanian University, P.O. Box 35247, Amman 11180, Jordan

## Abstract

The Mahalanobis Taguchi System (MTS) is considered one of the most promising binary classification algorithms to handle imbalance data. Unfortunately, MTS lacks a method for determining an efficient threshold for the binary classification. In this paper, a nonlinear optimization model is formulated based on minimizing the distance between MTS Receiver Operating Characteristics (ROC) curve and the theoretical optimal point named Modified Mahalanobis Taguchi System (MMTS). To validate the MMTS classification efficacy, it has been benchmarked with Support Vector Machines (SVMs), Naive Bayes (NB), Probabilistic Mahalanobis Taguchi Systems (PTM), Synthetic Minority Oversampling Technique (SMOTE), Adaptive Conformal Transformation (ACT), Kernel Boundary Alignment (KBA), Hidden Naive Bayes (HNB), and other improved Naive Bayes algorithms. MMTS outperforms the benchmarked algorithms especially when the imbalance ratio is greater than 400. A real life case study on manufacturing sector is used to demonstrate the applicability of the proposed model and to compare its performance with Mahalanobis Genetic Algorithm (MGA).

## 1. Introduction

Classification is one of the supervised learning approaches in which a new observation needs to be assigned to one of the predetermined classes or categories. If the number of the predetermined classes is more than two, it is a multiclass classification problem; otherwise, the problem is known as the binary classification problem. At present, these problems have found applications in different domains such as product quality [1] and speech recognition [2].

The classification accuracy depends on both the classifier and the data types. The classifier types can be categorized according to supervised versus unsupervised learning, linear versus nonlinear hyperplane, and feature selection versus feature extraction based approach [3]. On the other hand, Sun et al. [4] reported that the parameters affecting the classification are the overlapping between data (i.e., class separability), small sample size, within-class concept (i.e., a single class may consist of various subclasses, which do not necessary have the same size), and the data distribution for each class. If the data distribution of one class is different from distributions of others, then the data is considered imbalance. The border that separates balance from imbalance data is vague; for example, imbalance ratio, which is the ratio between the major to minor class observations, is reported from small values of 100 to 1 to 10000 : 1 [5].

The assumption of an equal number of observations in each class is elementary in using the common classification methods such as decision tree analysis, Support Vector Machines, discriminant analysis, and neural networks [6]. Imbalance data occurs often in real life such as text classification [7]. The problem of treating the applications that have imbalance data with the common classifiers leads to bias in the classification accuracy (i.e., the predictive accuracy for the minority class will be much less than for the majority class) and/or considering the minority observation as noise or outliers, which will result in ignoring them from the classifier.

To handle the classification of imbalanced data problem, the research community uses data and algorithmic or both approaches. For the data approach, the main idea is to balance the class density randomly or informatively (i.e., targeted) either eliminating (downsampling) the majority class observations or replicating (oversampling) the minority class observations or doing both. While at the algorithmic approach, the main idea is to adapt the classier algorithms towards the small class, a combination of the data and algorithmic levels approaches is also used and known as cost-sensitive learning solutions.

The problems reported [4] using data approach are as follows: deleting significant information for certain instances in case of downsampling, bringing noise to original data in case of oversampling, determining the appropriate sample size in within-class concept data, specifying the ideal class distribution, and using clear criteria for selecting samples.

While the problem reported [4] using the algorithmic approach is that it needs a deep understanding about the classier used itself and the application area (i.e., why a classifier deteriorates when imbalance data occurs).

Finally, the problem in using the cost-sensitive learning approach is the assumption of previous knowledge for many errors types and imposing a higher cost to the minority class to improve the prediction accuracy. Knowing the cost matrices in most cases is practically difficult.

While data and algorithmic approaches constitute the majority efforts in the area of imbalanced data, several other approaches have also been conducted, which will be reviewed in Literature Review.

To overcome the pitfalls of data and algorithmic approaches to solve the problem of imbalanced data classification, the classification algorithm needs to be capable of dealing with imbalance data directly without resampling and should have a systematic foundation for determining the cost matrices or the threshold. One of the promising classifiers is the Mahalanobis Taguchi System (MTS), which has shown good classification results for imbalance data without resampling, it does not require any distribution assumption for the input variables, and it can be used to measure the degree of abnormality (i.e., the degree of abnormality is proportional to the magnitude of Mahalanobis Distance for the positive observations), but unfortunately it lacks a systematic foundation for threshold determination [8].

The Receiver Operating Characteristics (ROC) based approach has been reported in the research domain [9] for Support Vector Machines (SVMs) and random forests (RF) as a cost function to trade off the required metrics (i.e., sensitivity versus specificity). Three operating point selection criteria, shortest distance, harmonic mean, and antiharmonic mean, have been compared, and the results in [9] showed that there is no difference among classifiers performances. Based on that, and up to author knowledge, no previous work has been reported for using ROC based approach to find the optimum threshold for the Mahalanobis Taguchi System (MTS) approach; therefore, a Modified Mahalanobis Taguchi System (MMTS) methodology is proposed in this paper.

The aim of this work is to enhance the Mahalanobis Taguchi System (MTS) classifier performance by providing a scientific, rigorous, and systematic method using the ROC curve for determining the threshold that discriminates between the classes.

The organization of the paper is as follows: Section 2 reviews the previous work of imbalance data classifications methods, the Mahalanobis Taguchi System, and its applications. In Section 3, the proposed Modified Mahalanobis Taguchi System (MMTS) methodology is described. In Section 4, results are presented for the comparison among the suggested MMTS algorithm with the Probabilistic Mahalanobis Taguchi System (PMTS), Naive Bayes (NB), and Support Vector Machine (SVM) through several datasets.  Section 5 presents a case study to demonstrate the applicability of the proposed research. And in Section 6, the results obtained from this research are summarized.

## 2. Literature Review

In this section, an overview of the imbalance classification approaches, the Mahalanobis Taguchi System concept, its different areas of applications, weakness points, and its variants is presented.

Solutions to deal with the imbalanced learning problem can be summarized into the following approaches [10]: sampling (sometimes called the data level approach), algorithmic, and cost-sensitive approaches.

Data level approach [11] is mainly returning the balance distribution between the classes through resampling techniques. It includes the following types:

1. Random undersampling∖oversampling of the negative∖positive observations
2. Targeted undersampling∖oversampling of the negative∖positive observations
3. Mixing approach from the above two items

The problems reported in data approaches are as follows:

- Determining the best class distribution or imbalance ratio for given observations: in Weiss and Provost [12], the relation between the classifier performance and the class distribution had been investigated; the results showed that balanced class distribution does not necessary produce optimal classification performance.
- Undersampling the negative data can lead to loose important information, whereas oversampling the positive one may cause noise interference [13].
- The uncertain criterion for selecting samples for within-class concept: that is, the class itself consists of several subclasses (i.e., how oversampling and/or undersampling will be performed for within-class concept).

Algorithmic level approach solutions are based upon creating a biased algorithm towards positive class. The algorithmic level approach has been used in many popular classifiers such as decision trees, Support Vector Machines (SVMs), association rule mining, back-propagation (BP) neural network, one-sample learning, active learning methods, and the Mahalanobis Taguchi System (MTS).

The adaptation of decision tree classifier to suit the imbalance data can be accomplished by adjusting the probabilistic estimate of the tree leaf or developing new trimming approaches [14].

Support Vector Machines (SVMs) showed good classification results for slightly imbalanced data [15], while for highly imbalanced data researchers [16, 17] reported poor performance classification results, since SVM try to reduce total error, which will produce results shifted towards the negative (majority) class. To handle the imbalance data, there are proposals such as using penalty constants for different classes found in Lin et al. [18] or changing the class border based on kernel adjustment as in Wu and Chang [19].

Therefore, in this paper, SVM was selected as one of the benchmarked algorithms to compare with ours; the results showed that SVM classification performance largely degrades with a high imbalance ratio, which supports the previous findings of the researchers (more details will be presented in Results).

Association rule mining is a recent classification approach combining association mining and classification into one approach [20–22]. To handle the imbalance data, determining many minimal supports for different classes to present their varied recurrence is required [23].

On the other hand, one-class learning [24, 25] used the target class only to determine if the new observation belongs to this class or not. BP neural network [26] and SVMs [27] are examined as one-class learning approach. In the case of highly imbalanced data, one-class learning showed good classification results [28]. Unfortunately, one-class learning algorithms drawbacks are that the size of the training data is relatively larger than those for multiclass approaches, and it is also hard to reduce the dimension of features used for separation.

Active learning approach is used to handle the problems related to the unlabeled training data. Research on active learning for imbalance data reported by Ertekin et al. [29] is based on the iterative approach by training the classifier on the data near the classification boundary instead of the whole training dataset, since the imbalance ratio for the dataset near the boundary is different from those away from the boundary. Unfortunately one of the bit falls for using this approach is that it can be computationally expensive [30].

The problem with the algorithmic approach is that it needs an extensive knowledge of specific classifier (i.e., why the algorithm fails to detect the positive cases), also understanding the application domain is critical (i.e., the effect of misclassification on the domain).

Cost-sensitive methods use both data and algorithmic approaches, where the objective is to optimize (i.e., minimize) the total misclassification cost while giving a positive class a higher misclassification cost [31, 32].

Cost-sensitive methods used different costs or penalties for different misclassification types. For example, let *C*_pos,neg_ be the cost of wrongly classifying positive instant as a negative one, while *C*_neg,pos_ is the cost of the contrary case. In imbalance data classification, usually, the revealing of the positive instant is more important than the negative one; hence, the cost of positive instance misclassification outweighs the cost of negatives ones (i.e., *C*_pos,neg_ > *C*_neg,pos_), with correct classification cost equal to zero (i.e., *C*_pos,pos_ = *C*_neg,neg_ = 0).

Different types of cost-sensitive approaches have been reported in the literature:

- Modifying the weights of the data space: in this approach, modification to the training data density is performed using the misclassification cost criteria, in a way that the density is adjusted towards the costly class.
- Making the classifier objective cost-sensitive: instead of minimizing the misclassification error, the objective is tuned to reduce the misclassification cost [32].
- Using risk minimization approach: in a binary c4.5 (i.e., decision tree) classifier, the assignment of a class type to a leaf end is based on the high-frequency class that reaches the end, while for the cost-sensitive classifier, the assignment of the class label is based on minimizing the classification cost [33].

The problem of using the cost-sensitive approach is that it is based on previous knowledge of the cost matrix for the misclassification kinds, while in most cases it is unavailable.

### 2.1. Mahalanobis Taguchi System (MTS)

MTS is a multivariate supervised learning approach, which aims to classify new observation into one of the two classes (i.e., healthy and unhealthy classes). MTS was used previously in predicting weld quality [3], exploring the influence of chemicals constitution on hot rolling manufactured products [34], and selecting the significant features in automotive handling [35]. The MTS approach starts with collecting considerable observations from the investigated dataset, tailed by separating of the unhealthy dataset (i.e., positive or abnormal) from the healthy (i.e., negative or normal). Calculation of the Mahalanobis Distance (MD) using the negative observation is performed first, followed by scaling (i.e., dividing the MD calculated over the number of features used), which will result in an average MDs around one for the negative observations. The scaled MD for the positive date set supposes to be different from MD for those for the negative dataset. Since many features are used to calculate the MD, so that the probability to have significant features for the multivariable dataset is high, Taguchi orthogonal array is used to screen these features. The criterion for selecting the appropriate features is determined by selecting the features that possess high MD values for the positive observations. It is worth noticing that a continuous scale is constructed from the single class observations by using MTS; unlike other classification techniques, learning is done directly from the positive and negative observations. This characteristic helps the MTS classifier to deal with the imbalance data problems.

The step of determining the optimal threshold is a critical one for effective MTS classier. To determine the appropriate threshold, loss function approach was proposed by [36]; however, it is not a practical approach because of the difficulty in specifying the relative cost [37]. In order to overcome this problem, Su and Hsiao [6] used a Chebyshev's theorem to specify the threshold and called their method a “probabilistic thresholding method (PTM)” for the MTS, whereas in MTS the threshold is assumed to be one. It has been shown in [6] that PTM classifier performance outperformed MTS classifier performance; therefore, it has been selected to be benchmarked with the proposed classifier. Unfortunately, the PTM method is based on previously assumed parameters, and the accuracy of the classification results was less than the benchmarked classifiers (this is one of the findings in this research, which will be discussed in Results).

The other research area in the MTS is related to the modification of the Taguchi method not in the threshold determination. Due to the lack of a statistical foundation [37] for the Taguchi method, the Mahalanobis Genetic Algorithm (MGA) [3] and the Mahalanobis Taguchi System using Particle Swarm Optimization (PSO) [38] have been used. Both the MGA and MTS Particle Swarm Optimization methods deal with the Taguchi system (orthogonal array) part, while the threshold determination still lacks a solid foundation or is hard to be determined in reality.

Finally, the aim of this research is to enhance the Mahalanobis Taguchi System (MTS) classifier performance through providing a scientific, rigorous, and systematic method of determining the binary classification threshold that discriminates between the two classes, which can be applied to the MTS and its variants (i.e., MGA).

## 3. Modified Mahalanobis Taguchi System (MMTS)

The proposed model, Algorithm 1, provides an easy, reliable, and systematic way to determine the threshold for the Mahalanobis Taguchi System (MTS) and its variants (i.e., Mahalanobis Genetic Algorithm, MGA) to carry out the classification process effectively. The currently used approaches either are difficult to use in practice such as the loss function [36] due to the difficulty in evaluating the cost in each case or are based on previously assumed parameters [6].

**Figure 1:**
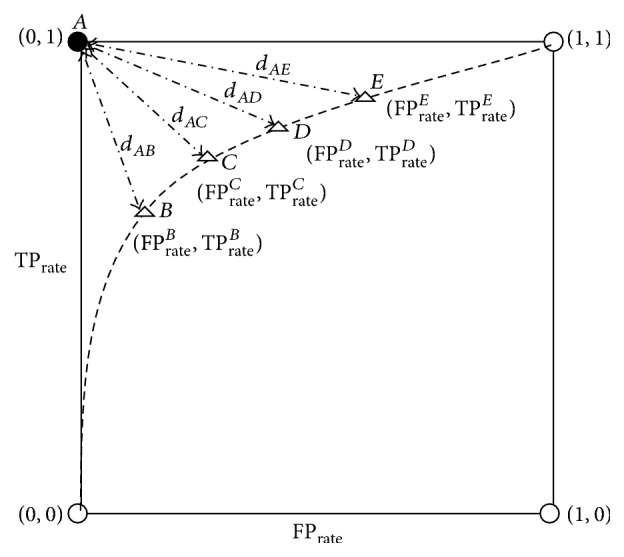
Receiver Operating Characteristics (ROC) curve for MTS.

The proposed model is based on using the Receiver Operating Characteristics (ROC) curve [39] for the MTS threshold determination. As shown in Figure 1, point *A* (TP_rate_ = 1, FP_rate_ = 0) represents the optimum theoretical solution (best performance) for any classifier. The closer the classifier performance to this point is, the better it is. The curve drawn in the figure represents the MTS classifier performance for different threshold values. Changing the threshold will change the point location on the curve (i.e., points *B*, *C*, *D*, and *E*). Therefore, the problem of finding the optimum threshold can be reformulated into the problem of finding the closest point that lies on the curve to point *A* (FP_rate_ = 0, TP_rate_ = 1).

MMTS can be summarized in the following steps.

**Step 1 (construction of the initial model stage).**

Assume there are two classes: negative (the one with majority observations) and positive (the one with the minority observations). A set of data is sampled from both classes. Using the negative observations only, reference Mahalanobis Distances are calculated using (1) with all features used. The Mahalanobis Distances (MD) for the positive observations are also calculated by using the same equation with all features, with the inverse of the correlation matrix of the negative observation used. Selection of the new features is performed by using the orthogonal array approach; then a recalculation of MDs for the negative and the positive observation is performed. An arbitrary threshold is assumed (i.e., one), and accordingly the true positive rate, the true negative rate, and the fitness function can be estimated.

**Step 2 (optimization stage).**

If the stopping criteria (i.e., fitness function value is zero, the number of maximum iterations is reached, and/or the differences among successive fitness value are less than a certain value) are not met yet, an optimization model (i.e., genetic algorithm) is invoked to obtain a better threshold value that minimizes the desired fitness function. Accordingly, new features will be selected using the orthogonal array approach, and true positive rate, false positive rate, and the fitness function will be also updated.

If the stopping criteria are met, then the training stage is done, and the model is ready for testing observations.

**Step 3 (testing stage).**

In this stage, the optimum threshold and the associated features are determined from the previous stage and the Mahalanobis Distance for the new observation is calculated based on those parameters. If the Mahalanobis Distance for this observation is less than the optimum threshold, then it will be classified as negative; otherwise, it will be classified as positive.

Now, after providing an overview of how MMTS algorithm works, detailed calculation of the Mahalanobis Distance, the true positive and the negative rates, and the fitness function will be presented in the followings subsection.

### 3.1. Mahalanobis Distance (MD)

In order to demonstrate the MTS threshold determination mathematically, let us assume that negative data (also called healthy or normal observations) and the positive data (also called unhealthy or abnormal observations) are available, where the number of positive observations is *N*_*p*_ and the number of negative observations is *N*_*n*_, and both positive and negative observations consist of *k* variables (or features).

Given a sample of size *N*_*n*_, the Mahalanobis Distance (MD) for the *i*th observation can be calculated by

(1) $$\begin{array}{c} MD_{i}=D_{i}^{2}=\frac{1}{k}Z_{ij}^{T}R^{-1}Z_{ij}, \end{array}$$

where *i* = 1 ⋯ *N*_*n*_,  *j* = 1 ⋯ *k*, *k* is total number of features (or variables), *Z*_*ij*_ is the normalized vector obtained by normalizing the values of *y*_*ij*_: that is, $Z_{ij}=(y_{ij}-{\bar{y}}_{j})/S_{j}$, where ${\bar{y}}_{j}$ and *S*_*j*_ are the average and the sample standard deviation of variable *j*, respectively, *Z*_*ij*_^*T*^ is the transpose of observation *i* and variable *j* for *Z*_*ij*_, and *R*^−1^ is the inverse of the correlation matrix of the negative variables.

Using (1), *R*^−1^, ${\bar{y}}_{j}$, *S*_*j*_, the inverse of the correlation matrix, the mean, and the sample standard deviation of the feature *j*, for the negative data, respectively, the MD of the positive observations can be calculated.

The next step is to determine the threshold *x* that will be used to discriminate the negative observations from the positive ones based on the MD magnitude, which means that the new observation *i* can be classified into either a positive or negative observation according to the following criteria:* if *MD_*i*_ < *x*, the observation is negative; otherwise, it is positive.

The contribution of this paper mainly is in the area of establishing a reliable and systematic threshold for classification. A rough method for determining the threshold is to plot the positive and negative MD observations versus their orders and decide upon the threshold manually. This method is not accurate, especially when dealing with the overlapping values of the MDs.

### 3.2. Proposed Threshold Determination

The essential classifier performance can be explained by examining the confusion matrix Table 1. The ratio between negative to positive observations (left to right columns in Table 1) is representation for the class distribution (i.e., imbalance ratio). In that sense, any performance metrics using both columns will be sensitive to the imbalance data issue, such as accuracy and error rate, (14) and (15), respectively. To overcome this problem, the Receiver Operating Characteristic (ROC) curves are recommended by the research community.

From the confusion matrix, Table 1, the following can be defined:

- TN^(*x*)^ is the total number of observations classified as negative from the pool of the negative observations (i.e., the negative observations whose MD < *x*).
- FN^(*x*)^ is the total number of observations classified as negative from the pool of the positive observations (i.e., the positive observations whose MD < *x*).
- FP^(*x*)^ is the total number of observations classified as positive from the pool of the negative observations (i.e., the negative observations whose MD ≥ *x*).
- TP^(*x*)^ is the total number of observations classified as positive from the pool of the positive observations (i.e., the positive observations whose MD ≥ *x*).

Now, the true positive rate and the false negative rate at the threshold *x* can be defined as

(2) TPratex=TPxNp,

(3) FPratex=FPxNn.

Using TP_rate_^(*x*)^ and FP_rate_^(*x*)^ for different values of threshold *x*, the ROC for the MMTS can be constructed.

The ROC plot is an *X*-*Y* plot in which TP_rate_^(*x*)^ (2) is plotted on the vertical axis and FP_rate_^(*x*)^ (3) is plotted on the horizontal axis.

Since TP_rate_^(*x*)^ uses the right column in the confusion matrix and FP_rate_^(*x*)^ uses the left column in the confusion matrix, they are unaffected by the imbalance data problem. The ROC is beneficial because it provides a tool to show the advantages (represented by true positives) versus disadvantages (represented by false positives) of the classifier relating to data density.

Figure 1 represents MTS classifier ROC curve, created by changing the MTS threshold (i.e., each point on the curve such as *B*, *C*, and *D* represents the different threshold for MTS classifier). The closest point lies on the curve (i.e., threshold) to point *A* (0, 1) which is considered the optimum threshold among the other candidates. Mathematically, this can be converted into the following optimization model.

#### 3.2.1. Nonlinear Optimization Model

The following optimization model is used to determine the optimum threshold *x* that discriminates between the negative and the positive observations, depending on minimizing the Cartesian distance between the MMTS ROC classifier curve and the theoretical optimum point (i.e., TP_rate_^(*A*)^ = 1, FP_rate_^(*A*)^ = 0).

(4) $$\begin{array}{c} d_{Ax}=\sqrt{{\left(FP_{rate}^{\left(A\right)}-FP_{rate}^{\left(x\right)}\right)}^{2}+{\left(TP_{rate}^{\left(A\right)}-TP_{rate}^{\left(x\right)}\right)}^{2}}, \end{array}$$

where  *d*_*Ax*_ is Euclidean distance between point *A* and any point *x* that lies on the ROC curve such as *B*, *C*, or *D*. FP_rate_^(*A*)^ is the false positive rate at point *A* which is equal to zero. TP_rate_^(*A*)^ is the true positive rate at point *A* which is equal to one. FP_rate_^(*x*)^ is the false positive rate at the threshold *x*. TP_rate_^(*x*)^ is the true positive rate at the threshold *x*.

Accordingly, the optimization model becomes

(5) minx dAx=FPrateA−FPratex2+PrateA−TPratex2,

(6) Subject  to: TPrateA=1,

(7)  FPrateA=0,

(8)  0≤FPratex≤1,

(9)  0≤TPratex≤1.

The optimization model is a nonlinear one, where the objective function is the Euclidean distance between points on the ROC MMTS curve and the “*A*” point (i.e., TP_rate_^(*A*)^ = 1, FP_rate_^(*A*)^ = 0). The first two constraints ((6) and (7)) are the theoretical optimum values of true∖false rate of the positive observations while the last two constraints (inequalities (8) and (9)) are the lower and the upper boundaries of the true positive rate and the false positive rate.

#### 3.2.2. Taguchi System

Since more features mean a higher cost of monitoring and require more processing time, it is important to exclude the unnecessary features from having an efficient classifier. MTS approach uses orthogonal array (OA) experiments to screen the important features. Each factor in the orthogonal array design can be calculated independently of all other factors since the design is balanced (i.e., the factors levels are weighted equally) (readers are referred to Woodall et al. [37] for further information about an OA).

The metric of the Taguchi orthogonal array is the signal-to-noise ratio, where *η* uses (in our case) “the larger the better” criterion, which can be calculated for different treatment *i* using

(10) $$\begin{array}{c} \eta_{i}=-10\log\frac{1}{N_{p}}\overset{N_{p}}{\underset{j=1}{\sum }}\frac{1}{MD_{i,j}^{2}}, \end{array}$$

where *i* is an index that represents run or row in the orthogonal design and its domain varied from 1 to 2^*k*^, where *k* is the total number of features. Based on the above equation, the feature mean gain can be calculated by

(11) Average  gainj=average  η  when  included  the  feature j−average  η  when  excluded  the  feature j,

where *j* is an index that represents the feature, *j* ∈ [1 ⋯ *k*], and *k* is the total number of features. The feature *j* will be included if it has a positive gain; otherwise, it should be excluded.

## 4. Results

In this section, the description of the dataset used in this study, brief of the used benchmarked classifiers, an overview of the metrics used for imbalanced data classifiers, and the results of classifiers performance for different datasets will be presented.

### 4.1. Dataset

The binary or multiclass imbalance ratio threshold, which is the ratio between negative to positive observations border that separates balance from imbalance dataset, is still an open area for the research community. In this paper, we investigated a wide range of IR, from 1.25 up to 2088, considering a dataset to be imbalanced if IR is equal or higher than 1.25.  Table 2 contains a description of the selected datasets properties. All the datasets (except for the welding dataset) were obtained from the UCI machine learning repository [41].

It should be noted in this study that the imbalance ratio effect on the classification results should be explored. Accordingly, the datasets were selected related to this criterion (i.e., to investigate at a wide range of IR). Unfortunately, imbalance ratio is not the only reason that causes degradation in classifier performance. The maximum Fishers Discriminant Ratio (*f*-ratio) is also considered as a major factor in classifier performance degradation. A low value of *f*-ratio means that observations are mixed together and overlapped regions are large, and therefore it is difficult to discriminate between these observations. Estimates of the different metrics were obtained by means of 10 repetitions; the data has been randomly partitioned by 35% as the training set and the remainder of the testing set for each repetition. MMTS and the benchmarked algorithms have been evaluated for each of the ten repetitions simultaneously.

### 4.2. Benchmarked Classifiers Used in the Study

In this section, an overview of the benchmarked classifiers, with their parameters, and the machine specifications used for analysis will be presented.

#### 4.2.1. Support Vector Machines (SVMs)

The first work regarding SVMs was published by Cortes and Vapnik [42], continued by significant contributions from other researchers [43]. SVMs showed a good classification performance for the rare and noisy data, which makes them favorable in a number of applications from cancer detection [44] to text classification [45].

**Figure 2:**
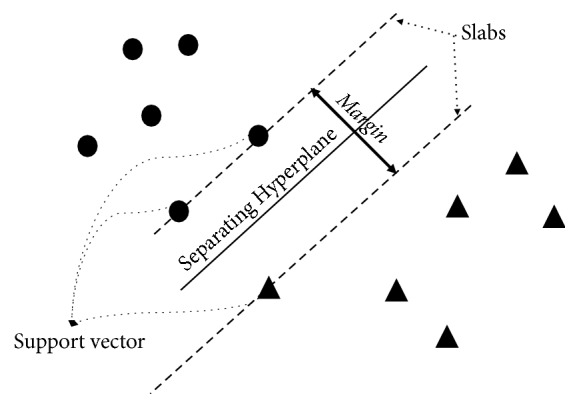
Supporting Vector Machines (SVMs).

The idea of the SVMs classifier is based on establishing the most appropriate hyperplane that separates class observations from each other (Figure 2). The most appropriate hyperplane means the one with the largest width of the margin parallel to the hyperplane with no interior points.

More details about SVMs methodology can be found in [46].

#### 4.2.2. Mahalanobis Taguchi System (MTS) Based on Probabilistic Thresholding Method (PTM)

In the PTM method, Chebyshev's theorem is employed to determine the threshold (12) that separates the normal observations from abnormal ones; see [6]:

(12) $$\begin{array}{c} x=\mu_{MDn}+\sqrt{\frac{1}{1+\delta -\omega }}\sigma_{MDn}, \end{array}$$

where *x* is the threshold that separates negative from positive observations, *μ*_MD*n*_ is the negative data mean MDs, *σ*_MD*n*_ is the negative data standard deviation MDs, *δ* is a small value, and *ω* is the portion of the negative observations whose MDs are less than the lower value of the positive MD observations.

#### 4.2.3. Naive Bayesian Classifier

Bayes theorem is the center of Naive Bayesian classifier (NB) in which class conditional independence is assumed. This assumption means that the influence of features on a given class is independent of each other. Mathematically,

(13) $$\begin{array}{c} P\left(\;X\;|\;C\;\right)=\overset{n}{\underset{i=1}{\prod }}P\left(\;x_{i}\;|\;C\;\right), \end{array}$$

where **X** = (*x*_1_, *x*_2_,…, *x*_*n*_) is a variable vector of size *n* and *C* is the class.

Even with such unrealistic assumption, Naive Bayes still found noticeable success stories comparable with other types of sophisticated classifiers, for example, NB used in text classification [47], medical diagnosis [48], and systems performance management [49].

### 4.3. Experimental Settings

The parameters values setting for the examined classifiers were selected from the suggestions of the corresponding authors as follows:

- MMTS: the MMTS does not need any tuning parameters, which is one of the important benefits of using MMTS over the traditional MTS.
- PTM: for the PTM algorithm, a small parameter is set to 0.05, based on the recommendation from [6].
- SVM: for the SVM algorithm, to map observations from the data space to the kernel space, the linear function was used.
- NB: for the NB algorithm, kernel distribution was selected to fit the conditional features distributions.

It is worth mentioning that no tuning parameters for any of the examined classifiers were performed; consequently, baseline line comparisons among the classifiers with the default setting were established, which leads to the most robust classifier selection [50].

Finally, MATLAB R2013a was used for the data analysis on HP machine with a processor Intel (R) Core (TM) i7 CPU 2.2 GHz and 4.00 GB RAM. For the genetic algorithm, the following parameters were used in the implementation: population size, 20 chromosomes, with the number of features corresponding to the bit number, 0.8, a crossover fraction, 0.01, a mutation rate, 100, and the limit for the number of generations, and for the stopping criteria, value of the fitness function cumulative change was less than 10–6 over 50 iterations.

### 4.4. Metrics

Several metrics such as accuracy (14), error (15), specificity (16), precision (17), sensitivity or recall (18), *G*_means_ (19), and *F*_measure_ (20) are used by the research community as comprehensive assessments of classifiers performances. The most important metrics among the above-mentioned ones are the sensitivity and the specificity, whereas the first one (sometimes called recall) can be seen as the accuracy of the positive observations: that is, how many positive observations were classified correctly. On the other hand, specificity can be understood as the accuracy of the negative observations: that is, how many negative observations were classified correctly.

Unfortunately, the examination of accuracy and error rates ((14) and (15)) reveals that these metrics are not sensitive to the data distribution [10]. For example, the given dataset consists of ninety percent of negative observations and ten percent of positive ones. If the classifier ignores the positives observations and classifies all instances as negative, it means that the classifier has ninety percent accuracy (i.e., error rate, 10 percent), which is a good classification performance for the entire dataset, but it cannot detect the positive instances as if it does not exist. In this context, it can be seen that accuracy and error rate metrics are biased towards one class on behalf of the other.

(14) AccuracyTN+TPNn+Np,

(15) Error1−Accuracy,

(16) SpecifictyTNTN+FP,

(17) PrecisionTPTP+FP,

(18) SensitivityRecall=TPTP+FN,

(19) GmeansSensitivity·Specificty=TPTP+FN·TNTN+FP,

(20) Fmeasure1+β2Recall·Precisionβ2Recall+Precision.

In order to overcome the above problem, several metrics such as *G*_means_ [51] (19), the area under a Receiver Operating Characteristic (AUC-ROC) curve [52], and *F*_measure_ [19] (20) are used to assess the imbalance data classifier performance.

The most common used metrics for the evaluation of the imbalance data classification performance are *G*_means_ and *F*_measure_, where the last one uses weighted importance of the recall and precision (controlled by *β*, the default value of *β* is 1), which results in better assessment than accuracy metric, but still biased to one class [10]. Therefore, *G*_means_ will be used as a main metric for the analysis criterion.

### 4.5. Classification Results

In this section, performance presentation for the classification results of MMTS with the other four investigated classification algorithms: Support Vector Machines (SVMs), Probabilistic Mahalanobis Taguchi System (PTM), Naive Bayes (NB), and Mahalanobis Taguchi System (MTS) (based on previously assumed threshold equal to one). In order to investigate the robustness performance of the studied classifiers related to the class imbalance criterion, fourteen different UCI [41] datasets and one data (welding) from El-Banna et al. [40] were used.

Table 3 summarizes the median values with the upper and the lower 95% confidence level interval based on nonparametric Wilcoxon Signed Rank Test for *G*_means_ values of the investigated data for the five classifiers. In order to discriminate between the classifiers performances among each other, nonparametric pairwise comparison Wilcoxon test was performed to test the null hypothesis that the two classifiers have equal medians versus the alternating hypothesis that the first classifier's median is larger than the second one; the results of these comparison are summarized in the ranking score of each classifier for each dataset. Based on this table, one can observe the following:

- The MMTS classifier has a higher classification performance than MTS across the whole fourteen investigated datasets.
- The MMTS has a superior classification performance comparable with the other benchmarked classifiers when the imbalance ratio (IR) is high (i.e., IR ≥ 463).
- The MMTS and SVM have equal classification performance when the imbalance ratio (IR) is medium (i.e., 189 ≤ IR ≤ 417).
- The SVM has a superior classification performance comparable with the other benchmarked classifiers when the imbalance ratio (IR) is low (i.e., 1 ≤ IR ≤ 189).
- The MMTS has the most robust classification performance over the investigated IR range (i.e., the MMTS ranks eight∖six times as the first∖second one, resp.).
- The NB has the least classification performance comparable with the other benchmarked classifiers over the investigated IR range.
- The effect of the *f*-ratio is dominated by the imbalance ratio (IR) effect (i.e., the IR is more important than the *f*-ratio).

#### 4.5.1. MMTS versus Modified SVMs and NB Classifiers

Many published works [16, 19, 53, 54] pointed out that SVMs classification performance drops significantly when dealing with the imbalance data; therefore, modified SVMs classifiers have been suggested to overcome this issue at both data and algorithmic levels. At the data level, Synthetic Minority Oversampling Technique (SMOTE) [11] has been applied successfully to handle the imbalance data issue, while at the algorithmic level, Adaptive Conformal Transformation (ACT) [54] and Kernel Boundary Alignment (KBA) [19] are among the most popular SVMs modified classifiers for imbalance data handling.

Therefore, in order to assess the MMTS classification performance against imbalance data classifiers, UCI datasets and their classification performance results using SVMs, SMOTE, ACT, and KBA from [19] were used, where the same experimental settings were used for the MMTS classifier in order to compare the benchmarked classifiers results.

Using the performance classification results obtained from [19] and the test performed using the MMTS classifier, *G*means performance metrics in the form of the 95% confidence intervals are reported in Table 4. It can be seen that the *G*means of the MMTS classifier are higher than those for the benchmarked classifiers at relatively high imbalance ratio (i.e., for the Abalone dataset), while for the yeast dataset, MMTS *G*means were less than KBA and ACT but better than SVM and SMOTE. Finally, MMTS was the least performance among the classifiers for the car dataset.

Using the same dataset in [19], modified NB algorithms such as tree augmented Naive Bayes (TAN), Hidden Naive Bayes (HNB), Average One-Dependence Estimators (AODE), and Weighted Average of One-Dependence Estimators (WAODE) are used to compare the MMTS classification performance with them.  Table 5 shows that the *G*means MMTS classification results for the examined datasets have the highest values comparable with the others.

## 5. Case Study

The case presented will be in the manufacturing sector in the area of resistance spot welding. Due to its cost and simplicity, resistance spot welding is the dominant joining process in the autoindustry. The reasons behind chosen spot welding joining process over other joining processes can be summarized as follows: being inexpensive and having fast process, its applicability to join different types of materials (coated steel, low carbon steel, aluminum, etc.) with varying thickness, and its relative robustness to the different noise factors existing in the plant such as fit-up variations. Despite the above-mentioned advantages, weld quality cannot be estimated with high certainty due to factors such as tip wear, sheet metal debris, variation in the power supply; therefore, it is common practice in the autoindustry to add extra welds to increase their confidence in the structural integrity of the welded assembly [40].

Recently worldwide competition pushes automotive OEMs to improve their productivity, reduce nonvalue added activity, and reduce cost. Therefore, autoindustry is extremely concerned with the elimination of these redundant welds. To achieve this objective of using the optimum number of required welds that sustain the required strength of the structure, weld quality must be achieved.

To achieve an acceptable weld quality, nondestructive weld assessment should be performed. This assessment can be translated into the problem of classifying the dynamic resistance profile (input signal) for those welds into normal or abnormal welds.

The welding data, summarized in Table 6, are used for this case having similar conditions to the one used in El-Banna et al. [40]. The experimental setup, the materials used, and all the other related information can be found in the same reference. The data consisted of 3,294 welds, from which 3,288 were normal welds, and the others were expulsion welds performed by an alternating current (AC) constant current controller. Each weld has 28 features, which represents the dynamic resistance value in the 28 half cycles or welding time. The welds were performed by an alternating current (AC) welding machine that has a capacity of 180 KVA with 680 lb of welding force provided by a pneumatic gun. An HWPAL25 truncated electrode type with a 6.4 mm face diameter was used with a welding time of 14 cycles and 11.3 KA as the initial input secondary current. Tip dressing was performed 10 times (approximately every 300 welds) in order to return the electrode tip to its original diameter by removing the excess material. The constant current control applied a current stepper, one Ampere per weld, to compensate for the increase in the electrode diameter or what is known as mushrooming effect.

### 5.1. Implementation

The first step after obtaining the dataset was to split them into training and testing groups. In this case, the training data was 1,153 observations (i.e., training ratio is 35%), in which two observations were expulsion welds (i.e., positive observations), and the others were normal welds (i.e., negative observations).

Running the MMTS and the other benchmarked algorithms, in addition to the Mahalanobis Genetic Algorithm (MGA) [3] over the welding data, Table 7 shows the results for the 10 repetitions in terms of the following metrics: specificity, sensitivity, precision, *G*_means_, and *F*_measure_. In addition, the suggested threshold is reported for the MMTS and PMTS algorithms. As mentioned before, *G*_means_ will be used as the main metric, but the results for other metrics will be reported here for future researchers to use.

In order to determine if there is a significant difference among the classifiers performances (i.e., *G*_means_), Table 7, nonparametric Kruskal-Wallis test is used, in which the *p* value obtained from performing this test on the welding data is 0.000, which reveals that there is at least one classifier performance that is significantly different from the others. In order to rank the classifiers, the pairwise Mann–Whitney test is used.

Table 8 shows the *p* values obtained from comparing the performances of the classifiers between any two classifiers using the Mann–Whitney test and the resulting classifiers rank. It can be seen clearly that the MMTS outperforms the other classifiers.

**Figure 3:**
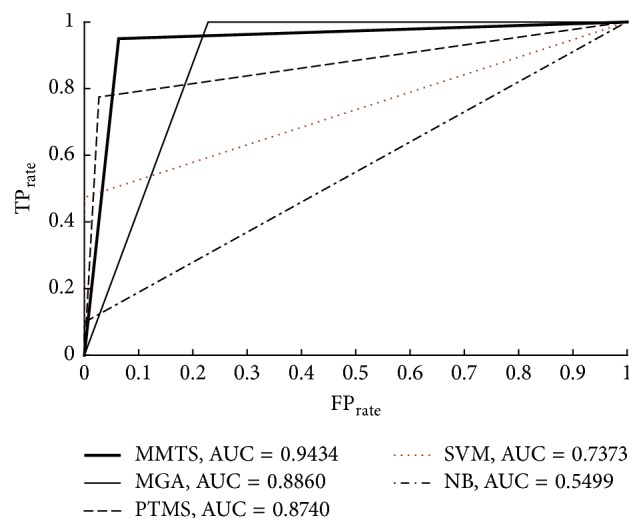
ROC curves for MMTS, PTM, SVMs, and NB classifiers for welding AC dataset.

This result is also emphasized in the ROC curves and the area under the curve (AUC) values for the examined classifiers (Figure 3).

## 6. Conclusions

The Mahalanobis Taguchi System (MTS) is one of the most promising binary classification approaches to handling the imbalance data problem. Unfortunately, the MTS suffers from the lack of a systematic rigorous method for determining the threshold to discriminate between the two classes. In this paper, a nonlinear optimization model with the objective of minimizing the Euclidean distance between MTS classifier ROC curve and the theoretical optimal point (i.e., TP_rate_ = 100% and FP_rate_ = 0%) is used to determine this threshold.

In order to assess the suggested algorithm, the MMTS has been benchmarked with several popular algorithms: Mahalanobis Taguchi System (MTS), Support Vector Machines (SVMs), Naive Bayes (NB), Probabilistic Mahalanobis Taguchi System (PTM), Synthetic Minority Oversampling Technique (SMOTE) with SVM, Adaptive Conformal Transformation (ACT), Kernel Boundary Alignment (KBA), Hidden Naive Bayes (HNB), and other improved Naive Bayes algorithms over benchmarked datasets with a wide range of imbalance ratio (i.e., 1.25 ≤ IR ≤ 2088). The results showed that the MMTS has a superior performance for high imbalance ratio (i.e., IR ≥ 463), while for the medium imbalance ratio (i.e., 189 ≤ IR ≤ 417), the MMTS has an equal classification performance with the SVMs. For the low imbalance ratio (IR ≤ 189), the SVM was the best among the classifiers. It has been noticed that the effect of the maximum Fishers Discriminant Ratio (*f*-ratio) is dominated by the imbalance ratio (IR) effect (i.e., IR is more important than *f*-ratio). MMTS showed a very robust classification performance across the range of the imbalance ratio; it also showed better classification performance results comparable with KBA, ACT (i.e., state of the art Modified SVM classifiers for imbalance data), HNB, NBtree, and other modified Naive Bayes classifiers when imbalance ratio is relatively high.

In order to demonstrate the MMTS applicability, a case study in the welding area was used. The results showed that the MMTS classifier performance outperformed the benched marked classifiers performances and MGA. The case results emphasize that the MMTS is one of the most suitable classifier algorithms when there is a high imbalance ratio.

For future research work, the problems of multiclass imbalanced data and the mixed data need to be tackled thoroughly.

## Figures and Tables

**Algorithm 1:**
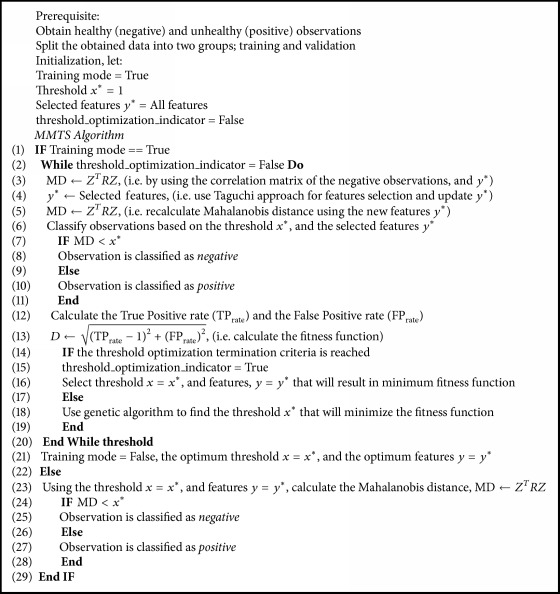
Modified Mahalanobis Taguchi System (MMTS) pseudo code.

**Table 1:** Confusion matrix.

		True class	
		Negative	Positive
Hypothesis output	Negative	TN^(*x*)^	FN^(*x*)^
	Positive	FP^(*x*)^	TP^(*x*)^
	Sum	*N*_*n*_	*N*_*p*_

TN^(*x*)^: true negative, FN^(*x*)^: false negative, FP^(*x*)^: false positive, TP^(*x*)^: true positive, based on threshold *x*, *N*_*n*_: negative observations, and *N*_*p*_: positive observations.

**Table 2:** Summary of the dataset used in the study.

Number	Dataset	Class	# variables	Number of observations		*f*-ratio^a^	IR ratio ^b^	*p* value^c^	Statistically
		Major/minor		Negative	Positive				Significant^d^
(1)	Abalone	Remainder/Class 24	8	4175	2	7.797	2088 : 1	0.0000	Yes
(2)	Abalone	Remainder/Class 22	8	4171	6	0.814	695 : 1	0.0000	Yes
(3)	Abalone	Remainder/Class 23	8	4168	9	0.661	463 : 1	0.0000	Yes
(4)	Abalone	Remainder/Class 3	8	4162	10	8.227	417 : 1	0.0028	Yes
(5)	Abalone	Remainder/Class 21	8	4165	12	1.244	347 : 1	0.0000	Yes
(6)	Abalone	Remainder/Class 21	8	4163	14	1.000	297 : 1	0.0000	Yes
(7)	Abalone	Remainder/Class 21	8	4151	22	1.019	189 : 1	0.0000	Yes
(8)	Abalone	Remainder/Class 21	8	4151	26	0.868	160 : 1	0.0000	Yes
(9)	Abalone	Remainder/Class 19	8	4145	32	0.555	130 : 1	0.0000	Yes
(10)	ECOLI	Remainder/Class OML	7	331	5	56.509	66 : 1	0.0000	Yes
(11)	Welding^e^	Normal/Expulsion	28	316	6	18.837	53 : 1	0.0122	Yes
(12)	Yeast	Remainder/Class ME2	8	1433	51	1.144	28 : 1	0.0000	Yes
(13)	Shuttle	Remainder/Class 5	9	41042	2458	11.513	17 : 1	0.0000	Yes
(14)	Glass	Remainder/Class 7	9	185	29	2.806	6 : 1	0.8156	No
(15)	Heart disease	Absence/Presence	13	150	120	0.872	1.25 : 1	0.0000	Yes

^a^Fisher discriminant ratio; data overlapping index, ^b^imbalance ratio = Negative/Positive; ^c^based on Kruskal-Wallis nonparametric test; ^d^is there any statistical significant difference among classifiers performance (yes/no)? ^e^[40].

**Table 3:** Summary of the classifiers performance ranks for all datasets.

Number	Dataset	*f*-ratio^a^	IR ratio^b^	MTS *G*_means_			MMTS *G*_means_			PTM *G*_means_			SVM *G*_means_			NB *G*_means_			Classifier rank				
				LL^h^	Med.^h^	UL^h^	LL	Med.	UL	LL	Med.	UL	LL	Med.	UL	LL	Med.	UL	MTS^c^	MMTS^d^	PTM^e^	SVM^f^	NB^g^
(1)	Abalone	7.797	2088 : 1	88.02	88.91	89.52	98.31	98.77	99.22	00.00	49.20	98.40	00.00	00.00	00.00	00.00	00.00	00.00	2	1	2	3	3
(2)	Abalone	0.814	695 : 1	50.50	60.40	70.20	74.70	82.90	91.00	00.00	00.00	39.60	28.40	56.80	68.30	00.00	00.00	27.90	2	1	3	2	4
(3)	Abalone	0.661	463 : 1	56.80	61.90	67.00	72.58	75.65	76.54	21.20	42.40	43.80	29.20	51.60	66.90	00.00	00.00	00.00	2	1	3	2	4
(4)	Abalone	8.227	417 : 1	84.14	84.58	85.43	96.63	97.33	97.92	28.70	57.30	77.80	81.30	90.00	98.70	69.70	80.50	94.40	2	1	3	1	2
(5)	Abalone	1.244	347 : 1	61.00	66.70	73.80	69.30	75.70	79.80	25.80	51.40	57.80	63.40	73.60	80.70	00.00	18.80	37.10	2	1	3	2	4
(6)	Abalone	1.000	297 : 1	64.40	69.90	75.50	71.54	76.18	81.03	32.20	51.50	60.30	63.50	74.00	85.20	23.50	38.70	45.30	2	1	3	1	4
(7)	Abalone	1.019	189 : 1	71.84	73.9	76.62	72.78	77.36	80.02	18.20	35.30	44.70	70.70	76.80	83.50	13.00	25.90	44.60	1	1	2	1	2
(8)	Abalone	0.868	160 : 1	69.06	71.84	74.68	73.82	78.15	81.35	29.40	44.50	56.30	81.78	83.30	85.18	24.20	38.90	48.50	3	2	4	1	4
(9)	Abalone	0.555	130 : 1	54.07	58.1	61.82	65.14	67.88	70.21	10.90	21.90	34.00	68.90	77.40	79.90	30.63	34.25	39.62	3	2	5	1	4
(10)	ECOLI	56.509	66 : 1	84.36	86.5	88.76	98.71	99.07	99.42	99.30	99.30	99.42	99.08	99.54	99.77	0.000	0.000	28.90	3	2	2	1	4
(11)	Welding^H^	18.837	53 : 1	57.80	65.00	71.70	79.70	89.10	98.30	79.60	86.10	92.70	38.90	69.00	89.20	38.00	67.50	84.50	2	1	1	2	3
(12)	Yeast	1.144	28 : 1	67.95	69.26	70.45	69.66	72.14	74.55	17.00	29.60	33.90	63.40	82.90	84.20	17.30	25.90	35.60	3	2	4	1	4
(13)	Shuttle	11.513	17 : 1	87.59	87.68	87.81	99.83	99.92	99.93	06.13	06.85	07.29	99.98	99.98	99.99	99.24	99.37	99.47	4	2	5	1	3
(14)	Heart	0.872	1.25 : 1	61.59	65.56	69.25	75.19	76.58	77.50	73.31	74.90	76.57	80.31	81.73	83.58	75.56	76.90	78.48	4	2	3	1	3

^a^Fisher discriminant ratio; data overlapping index; ^b^imbalance ratio = Negative/Positive; ^c^MTS: Mahalanobis Taguchi System classifier at threshold = 1; ^d^MMTS: Modified Mahalanobis Taguchi System classifier; ^e^PTM: Probabilistic Mahalanobis Taguchi System classifier; ^f^SVM: Support Vector Machines classifier; ^g^NB: Naive Bayes classifier; ^h^LL: lower limit, Med.: median, and UL: upper limit based on 95% confidence interval by using one sample Wilcoxon method; ^H^[40].

**Table 4:** Classification performance results (*G*means) of MMTS classifier versus modified SVMs class imbalance data classifiers.

Dataset	*f*-ratio	# variables	Number of observations		IR	SVM	SMOTE	ACT	KBA	MMTS
			Negative	Positive						
Car	1.01	6	1659	69	24 : 1	99.0 ± 2.2	99.0 ± 2.3	99.9 ± 0.2	99.9 ± 0.2	85.3 ± 2.2
Yeast	1.14	8	1433	51	28 : 1	59.0 ± 12.1	69.9 ± 10.0	78.5 ± 4.5	82.2 ± 7.1	72.2 ± 2.9
Abalone	0.55	8	4145	32	130 : 1	0.0 ± 0.0	0.0 ± 0.0	51.9 ± 7.6	57.8 ± 5.4	67.7 ± 3.4

**Table 5:** Classification performance results (*G*means) for the modified Naive Bayes classifiers.

Dataset	*f*-ratio	# variables	Number of observations		IR	HNB	TAN	NBTree	AODE	WAODE
			Negative	Positive						
Car	1.01	6	1659	69	24 : 1	74.8 ± 7.1	49.6 ± 11.5	52.2 ± 22.8	2.3 ± 7.3	8.6 ± 14.5
Yeast	1.14	8	1433	51	28 : 1	45.5 ± 26.9	34.9 ± 25.1	23.5 ± 25.5	4.1 ± 12.9	25.8 ± 22.8
Abalone	0.55	8	4145	32	130 : 1	0.0 ± 0.0	0.0 ± 0.0	0.0 ± 0.0	0.0 ± 0.0	0.0 ± 0.0

**Table 6:** Description of welding data.

Dataset	Classes	Number of vars.	Neg. obs.	Pos. obs.	*f*-ratio	IR
AC welding	Normal/expulsion	28	3288	6	4.1104	548 : 1

**Table 7:** Classification results for AC welding dataset with IR 548.

Classifier type	Repetition	Threshold (*x*)	Specificity	Sensitivity	Precision	*G*_means_	*F*_measure_
MMTS	1	4.661	99.392	75.000	99.195	86.339	85.417
	2	1.588	87.319	100.000	88.746	93.444	94.037
	3	6.415	99.345	75.000	99.134	86.318	85.395
	4	2.339	95.367	100.000	95.572	97.656	97.736
	5	1.858	91.015	100.000	91.756	95.402	95.701
	6	2.929	98.549	100.000	98.570	99.272	99.280
	7	2.789	98.315	100.000	98.343	99.154	99.165
	8	1.653	89.190	100.000	90.245	94.441	94.872
	9	1.254	79.551	100.000	83.023	89.191	90.724
	10	3.074	98.690	100.000	98.707	99.343	99.349

PTM	1	3.803	98.549	75.000	98.103	85.972	85.010
	2	3.718	98.737	75.000	98.343	86.054	85.100
	3	3.279	96.912	75.000	96.045	85.255	84.228
	4	2.312	95.087	100.000	95.317	97.512	97.602
	5	4.416	99.438	25.000	97.803	49.859	39.821
	6	2.503	97.099	100.000	97.181	98.539	98.570
	7	2.112	95.367	100.000	95.572	97.656	97.736
	8	4.775	99.064	50.000	98.163	70.379	66.253
	9	3.137	95.929	75.000	94.851	84.821	83.766
	10	2.492	96.912	100.000	97.004	98.444	98.479

SVM	1	—	99.953	25.000	99.813	49.988	39.985
	2	—	100.000	50.000	100.000	70.711	66.667
	3	—	99.953	75.000	99.938	86.582	85.691
	4	—	99.953	75.000	99.938	86.582	85.691
	5	—	100.000	25.000	100.000	50.000	40.000
	6	—	99.906	50.000	99.813	70.678	66.625
	7	—	99.906	50.000	99.813	70.678	66.625
	8	—	100.000	25.000	100.000	50.000	40.000
	9	—	99.953	75.000	99.938	86.582	85.691
	10	—	99.953	25.000	99.813	49.988	39.985

NB	1	—	100.000	25.000	100.000	50.000	40.000
	2	—	100.000	25.000	100.000	50.000	40.000
	3	—	99.906	0.000	0.000	0.000	NaN^a^
	4	—	100.000	25.000	100.000	50.000	40.000
	5	—	99.953	0.000	0.000	0.000	NaN
	6	—	100.000	0.000	NaN	0.000	NaN
	7	—	100.000	25.000	100.000	50.000	40.000
	8	—	100.000	0.000	NaN	0.000	NaN
	9	—	99.906	0.000	0.000	0.000	NaN
	10	—	99.953	0.000	0.000	0.000	NaN

MGA	1	1.000	77.164	100.000	81.410	87.843	89.752
	2	1.000	77.118	100.000	81.379	87.817	89.733
	3	1.000	76.977	100.000	81.286	87.737	89.677
	4	1.000	77.164	100.000	81.410	87.843	89.752
	5	1.000	76.837	100.000	81.193	87.657	89.621
	6	1.000	77.492	100.000	81.627	88.029	89.884
	7	1.000	77.211	100.000	81.441	87.870	89.771
	8	1.000	77.164	100.000	81.410	87.843	89.752
	9	1.000	77.164	100.000	81.410	87.843	89.752
	10	1.000	77.632	100.000	81.721	88.109	89.941

^a^NAN since the dominator is zero.

**Table 8:** Mann–Whitney test *P* values. ^a^ Results for welding AC dataset with IR 548.

		*G*_means_ Median_2_					Classifier rank
		MMTS	PTM	SVM	NB	MGA	
*G*_means_ Median_1_	MMTS	—	0.0410	0.0005	0.0001	0.0129	1
	PTM	—	—	0.0521	0.0003	**0.2405**	2
	SVM	—	—	—	0.0070	0.0001	3
	NB	—	—	—	—	—	4
	MGA^b^	—	**0.2363**	0.0001	0.0001	—	2

^a^The null hypothesis *H*_*o*_ : Median_1_ = Median_2_ is tested versus the alternative hypothesis *H*_1_ : Median_1_ > Median_2_, at a specified level of significance *α* = 0.05; ^b^Mahalanobis Genetic Algorithm [3].
